# Nutritional status and age at menarche in Amazonian students

**DOI:** 10.1016/j.jped.2024.03.002

**Published:** 2024-03-21

**Authors:** Kettyuscia Coelho e Oliveira, José Cardoso Neto, Davi C. Aragon, Sonir R. Antonini

**Affiliations:** a Universidade do Estado do Amazonas (UEA), Departamento de Clínica Médica, Manaus, AM, Brazil; b Universidade de São Paulo, Faculdade de Medicina de Ribeirão Preto, Departamento de Pediatria, Ribeirão Preto, SP, Brazil; c Universidade Federal do Amazonas (UFAM), Departamento de Estatística, Manaus, AM, Brazil

**Keywords:** Menarche, BMI, Race, Height, Socioeconomic level

## Abstract

**Objectives:**

Age at menarche (MA) is a proxy for biological maturation and a parameter of socioeconomic changes. Worldwide, anticipation of menarche is associated with nutritional transition and excess weight. The objective of this study was to evaluate the MA in Amazonian students and its association with nutritional status, ethnicity, and socioeconomic level.

**Methods:**

Cross-sectional study with 1,017 students aged 6 to 17 living in the city of Manaus, Brazil. MA was analyzed by status quo and recall; its association with body mass index (BMI), race, socioeconomic status, and adult height was examined.

**Results:**

559 (51.9%) participants had already experienced menarche. In 91.7%, menarche occurred between 10 and 14 years of age; the mean age at the onset of menarche was 11.9 years. Overweight (11.6 years) and obese (11.4 years) participants reached menarche earlier than those with normal weight (12 years) and lean (12.7 years) participants. The associations between MA and nutritional status showed that overweight and obesity are risk factors for the early occurrence of menarche. MA was not associated with socioeconomic status/parental education or race. However, excess weight was associated with earlier MA in all races and social classes. The adult height was slightly lower in girls with menarche before 12 years old (157.9 vs 159.4 cm).

**Conclusion:**

Regardless of socioeconomic level or ethnicity, excess weight was associated with earlier menarche in Amazonian students.

## Introduction

Monitoring secular trends in the pubertal development of a country's children is essential for social and public health reasons.^1^^,^^2^ The phenomenon described as the “secular trend in MA”, which demonstrates an anticipation of menarche, was first observed in Europe and the United States in the mid-19th century and was due, at least in part, to an increase in body mass index (BMI) during childhood.^3^ The timing of the onset of puberty has changed notably in recent centuries. Until the middle of the 20th century, MA gradually declined in many industrialized countries. Data from Europe show that MA decreased drastically throughout the 19th and 20th centuries. In the mid-19th century, menarche occurred at around 17 years of age. However, in the second half of the 20th century, it occurred around 13 years. At the end of the 20th century, this trend ceased in developed countries, probably due to stable socioeconomic conditions and improved nutritional and hygiene conditions. Since the 1960s, the downward trend of MA appears to have diminished in both Europe and the USA, but has remained significant, 2.5 to 4 months over the last 25 years.^4^^,^^5^^,^^6^^,^^7^

**Figure 1:**
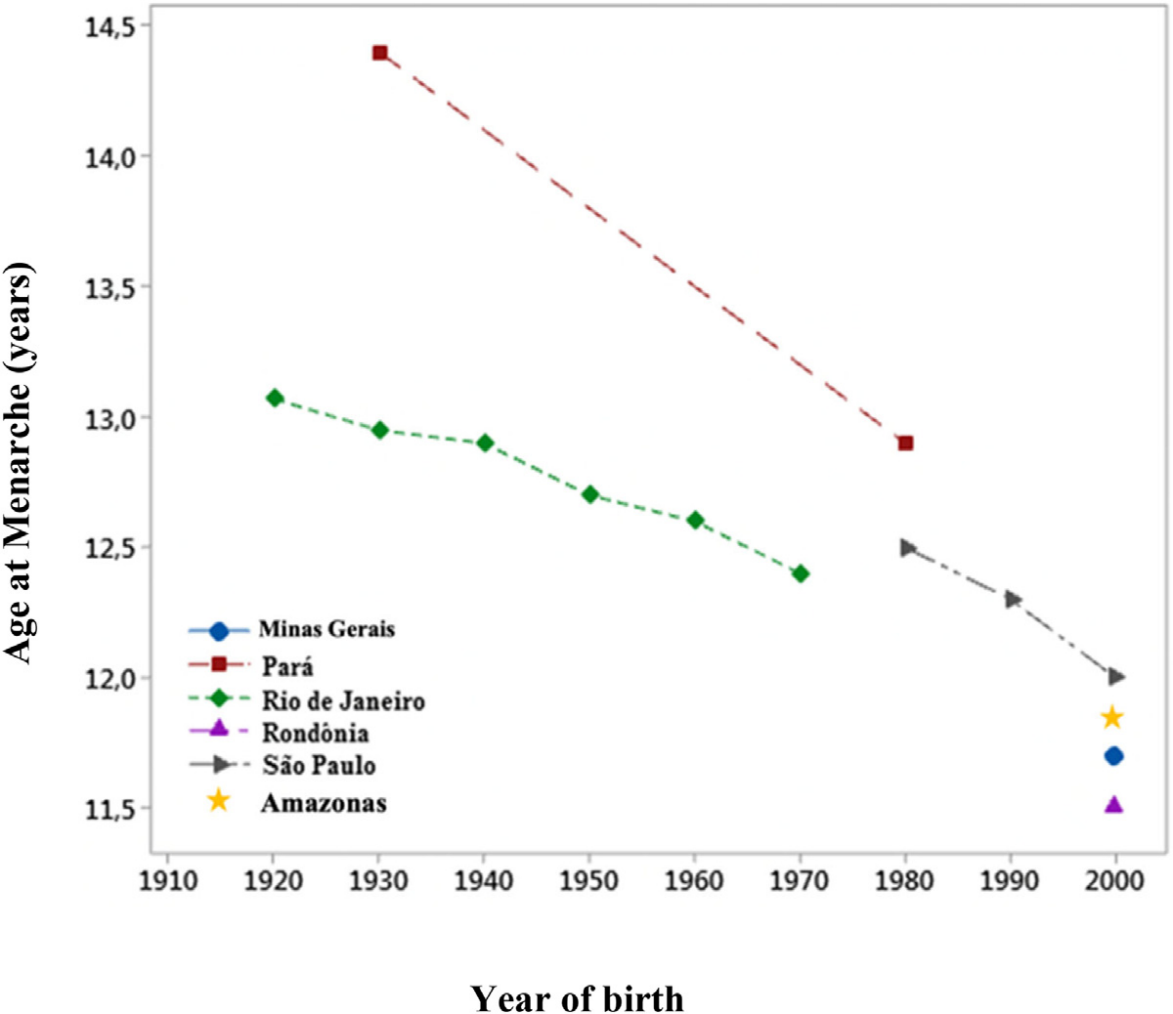
Trend of Secular Menarche in Brazil.

Source: Prepared by the Author.

In this century, in the first decade of the 2000s, reported MA in the USA was 12.2 years, corresponding to a three-month decrease over the last 20 years.^8^ The Copenhagen study showed that the age of onset of puberty decreased by 12 months between 1991 and 2006 (1991 cohort: 10.9 years; 2006 cohort: 9.9 years). However, in that population, the decrease in MA was much less significant: three months in the same period (1991 cohort: MA = 13.4 years; 2006 cohort: MA = 13.1 years).^9^ The trend of decreasing MA was also described in Brazil. An eight-month-earlier MA was reported among individuals born in the 1920s and 1970s.^10^ A trend study reported that menarche occurred 3.3 months earlier in 2010 than in 2001.^11^ In another Brazilian study, menarche occurred 19.4 months later in individuals born in 1930 (MA = 14.5 years) than in individuals born in 1980 (MA = 12.9 years).^12^ More recent studies have shown that this anticipation continues in the country, and MA under 12 years old has already been registered in many Brazilian regions (Figure 1).^2^^,^^10^^,^^11^^,^^12^^,^^13^

The objective of this study was to evaluate MA and its association with nutritional status, ethnicity, and socioeconomic status in a little-studied region of the Amazon, Manaus, capital of the state of Amazonas, northern Brazil, which has an estimated population of 2,130,264 people. It ranks 23rd among the 27 Brazilian capitals according to the Human Development Index (HDI). Most of the city's population is made up of mixed race (67.8%), followed by whites (26.6%), blacks (4.2%), yellow (1.1%). Indigenous people correspond to only 0.2% of the population.^14^

## Methods

A cross-sectional study was carried out with 1,017 students aged 6 to 17 from public and private schools in the urban area of ​​the city of Manaus. A probabilistic sample was stratified by type of school and age of participants, monitoring the distribution of students enrolled in public (75%) and private (25%) schools. The sample size was calculated to estimate the mean of a quantitative variable in a finite population, with a reliability coefficient of 95%. The sample size required for this study was 1,010 participants. Considering the possible losses of the study, the sample was overestimated by almost 100%, totaling 2,016 students. One public and one private school were drawn in each of the six geographic areas of the city of Manaus. This measure guaranteed the inclusion of participants from all areas of the city and of different ethnicities and socioeconomic conditions. Thus, the following were drawn: 1,512 students from public schools and 504 students from private schools in 12 schools (Supplementary Fig. 1). Data collection for the present study was carried out from June to October 2018.

The inclusion criteria were students aged between 6 and 17 years old, attending public or private schools, who were selected through a draw. Students with special needs that compromised anthropometry, pregnant teenagers, chronic illnesses, or those using medications (present or past) that could interfere with height, weight, and MA were excluded.

MA was determined using the *status quo* method. For those who had already reached menarche, the age and date (month and year) of the event were requested (recall method). The socioeconomic level was estimated by *proxy* through the type of school attended (public or private) and the level of education of the head of the family, divided into five categories: illiterate/incomplete elementary school I, complete elementary school I/incomplete elementary school II, complete elementary school II/incomplete high school, complete high school/incomplete higher education, and complete higher education. Race/skin color was assessed in a self-declared manner by the participant and guardian, in five categories: white, black, yellow, mixed race, and indigenous. To minimize possible discrepancies between current BMI and that at the time of menarche, a subsample of adolescents whose menarche occurred in the same year in which the study was carried out was analyzed. Therefore, in the girls of this group, the evaluation BMI occurred very close to the time of the menarche. Height and weight measurements were used to calculate BMI (BMI = Weight/Height^2^). BMI was assessed using the z-Score, and its classification based on the World Health Organization (WHO, 2007) criteria as thin (BMI z-score < −2), eutrophy (BMI ≥ *z*-score −2 and ≤ *z*-score +1), overweight (BMI > *z*-score +1 and ≤ *z*-score +2), obese (BMI > *z*-score +2). Weight was obtained using an AVANUTRI® GBF830 digital electronic scale (capacity of 180 kg) and height using an AVANUTRI® portable stadiometer (graduated to 200 cm). All these parameters were obtained by a single well-trained investigator (KCO) to minimize potential variability.

The age range was divided into one-year intervals. The occurrence of menarche was computed using the status quo method for each declared interval. Then, using a product limit estimator (Kaplan-Meier), the age at which 50% of participants had menarche was estimated, and the median MA was calculated. The MA mean was also calculated using the recall method, and a comparison was established between both methods (recall and *status quo*). The differences between the means were performed by fitting the linear regression model. Simple and multiple Cox proportional hazard models were used to calculate the crude and adjusted (considering race and socioeconomic level as covariates) hazard ratio, taking as a reference the category defined as eutrophy between MA and the BMI categorized. The software used was R 3.5.1, and a significance level of 5% was set for all analyses.

This research was approved by the Human Research Ethics Committee (CEP) of the State University of Amazonas, under n° 2,680,868. Authorization was requested from the Municipal and State Education Departments of Manaus and participating schools. An informed consent form (ICF) was required from those responsible, in addition to the assent form for participants over 12 years of age.

## Results

Of the 504 students from private schools and 1,512 students from public schools (2,016 students), 176 students (private schools) and 818 students (public schools) did not sign the ICF. Five of the remaining 1,022 students were excluded from the research because they used or had used medications that could affect growth or puberty. The final sample was 1,017 girls, 691 students from public schools (68%) and 326 students from private schools (32%).

**Figure 2:**
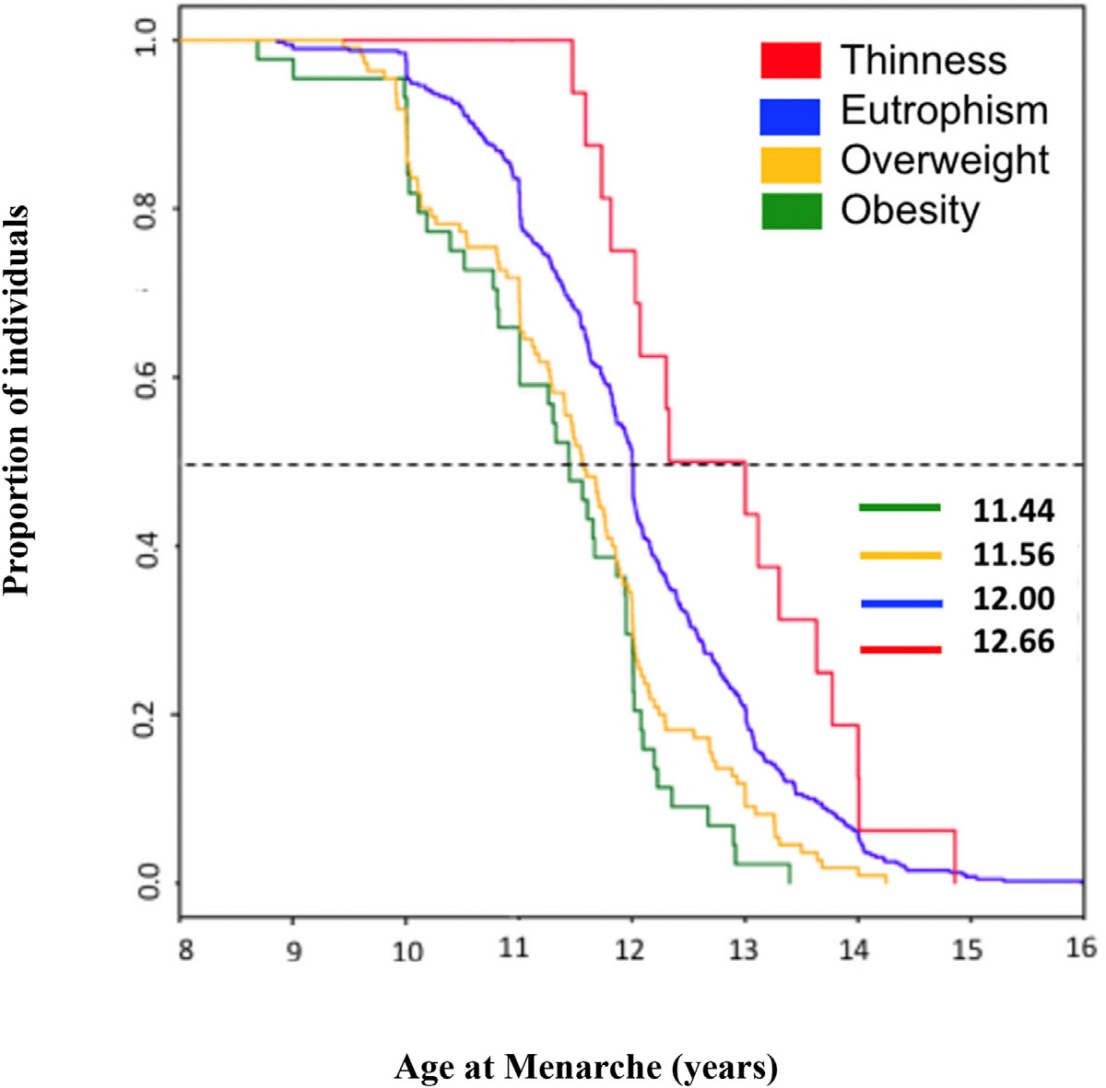
Median age at onset of menarche according to nutritional status of girls aged 6 to 17 years in Manaus, AM.

Menarche was reported by 559 (51.9%) of the participants. MA ranged from 8 to 16 years. The majority (61.8%) reported menarche between 11 and 13 years old (33.5% between 11 and 12 years old and 28.3% between 12 and 13 years old). Menarche occurred after the age of 14 in 5.1% and early menarche (MA < 10 years) in 3.2% of participants, 0.5% of whom menstruated before the age of 9. Using the *status quo* method, the median MA was 11.9 years. The mean MA using the recall method was also 11.9 [1.8] years.
The median MA (status quo) in lean (12.7 years), normal weight (12 years), overweight (11.6 years), and obese (11.4 years) participants were described in Figure 2 (*p* < 0.01).

The mean MA (recall) according to racial, socioeconomic, and nutritional categories is shown in Table 1. The authors only found an association between MA and nutritional status (*p* < 0.01) (Table 1). In the subgroup of girls whose menarche occurred in the same year in which the study was carried out (*n* = 70), implying that their BMI was measured closer to menarche, the mean [SD] MA in girls with obesity was 11.48 [0.67], overweight 11.62 [0.82], normal weight 12.01 [0.86].

**Table 1:** Distribution of the average age at menarche according to sociodemographic data and nutritional status of girls aged 6 to 17 years, Manaus, Brazil 2018.

Characteristics	Number of participants	Mean age at menarche (years) [SD]	(95%) CI	p-value
***Race/Skin color***				
White	**110**	**12.0** [1.2]	(11,76; 12,21)	
Black	**13**	**12.1** [1.3]	(11,38; 12,91)	
Mixed race	**426**	**11.8** [1.2]	(11,74; 11,96)	0.64
Yellow / Asian	**7**	**11.9** [1.6]	(10,35; 13,39)	
Indigenous	**3**	**11.4** [0.4]	(10,28; 12,46)	
***Education of the head of the family***				
Illiterate /Incomplete Elementary I	**21**	**11.6** [1.2]	(11,05; 12,15)	
Complete Elementary I /Incomplete Elementary II	**40**	**11.7** [1.0]	(11,37; 12,06)	
Complete Elementary II /Incomplete High school	**59**	**11.9** [1.4]	(11,56; 12,32)	0.23
Complete High School /Incomplete Higher	**209**	**11.8** [1.1]	(11,63; 11,93)	
Complete Higher Education	**171**	**12.0** [1.1]	(11,84; 12,17)	
***School Administration***				
Public	**402**	**11.9** [1.18]	(11,78; 12,01)	
Private	**157**	**11.8** [1.16]	(11,66; 12,03)	0.65
***Nutritional status***				
Thinness	**16**	**12.8** [1.04]	(12,26; 13,37)	
Eutrophic	**389**	**12.0** [1.15]	(11,90; 12,13)	
Overweight	**110**	**11.5** [1.11]	(11,29; 11,71)	< 0.01
Obesity	**44**	**11.3** [1.04]	(10,96; 11,59)	

In the present study, the prevalence of overweight was 17.4%, and obesity was 8.1% in the entire sample at the time of the study. The average median adult height at age 17 years was 159.7 cm.

In the Cox model, adjusted for age, the crude hazard ratio (95% CI) in obesity was 2.05 (1.49, 2.81), overweight 1.59 (1.28, 1.97), and in thinness 0.41 (0.10, 1.68). After adjusting for race and socioeconomic level as covariates, the hazard ratio (95% CI) in obesity was 2.17 (1.55, 3.03), overweight 1.66 (1.32, 2.08), and thinness 0.9 (0.09, 1.61).

**Figure 3:**
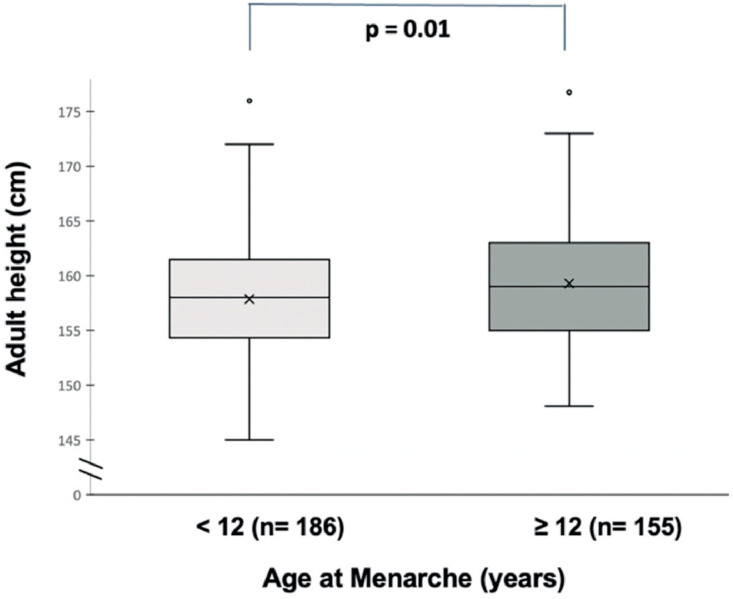
Near adult height in relation to the age at menarche in girls from Manaus, AM – Brazil (*n* = 341).

The authors analyzed how MA affected adult height in a subgroup of 341 participants who had menarche two or more years ago and were likely to have reached or approached their adult height. The authors observed that using the 12-year-old MA as a cutoff point, height was different between those with an MA less than or equal to 12 and those greater than 12 (157.9 cm vs. 159.4 cm, respectively; *p* = 0.01) (Figure 3). However, when the authors used the 10-year MA as a cutoff point, no difference in adult height was observed between the participants (158.9 cm vs 158.6 cm, respectively; *p* = 0.05).

## Discussion

The authors assessed MA and how it relates to nutritional status and adult height in girls born in the first decade of the 2000s in Manaus, the largest city of the Brazilian Amazon region. The mean and median MA was 11.9 years. MA was associated with BMI, not race, ethnicity, or socioeconomic level. Obese participants menstruated about 0.6 years (7 months) earlier than normal-weight participants and 1.3 years (16 months) earlier than lean participants. The associations between MA and nutritional status showed that overweight and obesity are risk factors for the occurrence of menarche.

The MA reported in this study is consistent with other Brazilian studies carried out in recent decades. An MA of 11.7 years was reported in Brazil in 2019;^15^ Another study showed 11.5 years in Rondônia^13^ and 11.7 years in Minas Gerais.^2^ MA trend data in Brazil show a decline in all regions over the decades: In Rio de Janeiro, MA decreased from 13.07 to 12.40 years between 1920 and 1979, representing an anticipation of 1.3 months per decade.^10^ In São Paulo, for those born between 1980 and 2000, there was a reduction of 3.3 months.^11^ Finally, in the Northern state of Pará, the same region where the present study was performed, MA decreased from 14.4 years to 12.9 years among those born in the 1930s and 1980s.^12^

The increasing global prevalence of obesity raises questions about the extent to which excessive weight may influence MA. Recent studies indicate a relationship between early puberty in girls and increased BMI.^11^^,^^13^^,^^15^, ^16^, ^17^ It has been widely reinforced that improved health and nutrition were responsible for most of this decline in MA.^3^ Although the question of whether early puberty is a cause or result of increased body fat has not been resolved, longitudinal studies suggested that increased body fat, or BMI, predicts the early onset of puberty.^8^^,^^18^^,^^19^ North American data demonstrated that MA decreased between the 1960s and 1990s, from 12.7 to 12.5 years. In the same interval, the percentage of individuals aged between 10 and 15 years who are overweight increased from 16% to 27% in that population.^6^ In China, there was a decrease in MA from 15.7 years in 1950 to 12.5 years in 2010. That country observed a decline in the MA of 4.5 months per decade in the last 25 years and an inverse association with BMI.^16^^,^^20^

The data on MA and the association between MA and BMI is comparable with other Latin American countries. In indigenous people of Argentina, MA was 11.6 years in the last decade.^21^ In the same country, MA was lower in overweight girls (11.5 years) than in normal-weight girls (12.1 years).^22^ In Bolivia, at the beginning of this century, MA in the rural population was 12.9 years, and the prevalence of overweight in women was 35%.^23^ In Colombia, among those born in the 1990s and 2000s, MA was 12.6 years, and an estimated decline of 0.5 years/decade. BMI, maternal education, and family wealth were inversely associated with MA.^24^ In Chile, in the first decade of this century, MA was 11.9 years, and 47% were overweight or obese.^25^

In Brazil, in the southwestern region of the Brazilian Amazon, the risk of menarche for each kg of fat was 1.04 times greater, and for each kg of muscle, it was 1.17 times greater.^17^ The same authors reported an MA of 11.5 years and a prevalence of overweight of 28.1% in the region.^13^ The association between excess weight and earlier MA was demonstrated; between 2001 and 2010, MA occurred approximately 3.2 months earlier. This decrease was greater in the group of overweight girls (5.8 months) than in those with normal and lean weight (1.4 months). In the same period, the prevalence of obesity increased from 4.5% in 2001 to 10% in 2010. The anticipation of menarche also occurred in the group of eutrophic women, indicating that other factors, in addition to excess weight, were contributing to this anticipation.^11^ A study with two birth cohorts in southern Brazil (1982 and 1993) demonstrated that the prevalence of menarche before the age of 11 was 24.7% (1982) and 27.6% (1993). Menarche before age 11 was positively associated with adiposity in adolescence and adulthood. However, after adjusting for BMI at age 11, the association between MA and body composition in adulthood was strongly related to body composition in late childhood.^26^

Overweight adolescents presented menarche earlier (11.3 years) than the Brazilian average (11.7 years). Individuals from Brazil's South and Southeast regions had MA earlier than other regions, 11.7 years. Interestingly, these two regions include the five wealthiest states and the highest rates of overweight and obesity in Brazil.^15^ These data agree with this study, where excess weight was associated with earlier MA in all races and social classes. In the present study, the authors also demonstrated the inverse association between MA and BMI when analyzing only girls whose menarche occurred in the same year. Also, in this sub-sample analysis, MA was lower among those who were overweight and higher among those who were normal weight or thin.

A previous study showed a tendency for MA to be earlier in black girls and more advanced in indigenous girls.^15^ However, the data did not confirm that finding. The authors did not observe differences in the MA between white and non-white girls. It is important to note that, although located in the Amazon region, the number of Indigenous girls was very low in this study. This distribution was in accordance with populational data showing that in the urban area of Manaus, Indigenous people account for less than 0.5% of the population.

A positive relationship between earlier MA and a higher socioeconomic level has been described by some authors.^15^^,^^27^^,^^28^ In the present study, assessing socioeconomic level through the level of education of the head of the family and the type of school attended, the authors did not find any association between MA and the socioeconomic level of the family, although girls with a head of the family with higher education presented later MA (12.0 years) than those with an illiterate head of the family (11.6 years). In line, MA was slightly lower in girls from private schools (11.8 years) than in public schools (11.9 years).

In the clinical scenario, the patient's parents frequently worry that a normal but early puberty results in lower adult height. The authors addressed this question in the present study by comparing the near-adult height in the group presenting menarche before and after 12 years. The authors observed that girls with menarche before 12 years presented a slight but significant lower adult height. Of note, this difference in height was only 1.5 cm.

Earlier MA has been associated with an increased risk of insulin resistance, type 2 diabetes mellitus, and cardiovascular disease in adulthood.^29^^,^^30^ Considering that obesity is identified as one of the most critical factors for the anticipation of MA, it is reasonable to assume that preventing obesity could reduce the risk of these diseases in adult life.

A limitation of the present study is the cross-sectional nature of the data. Therefore, it is impossible to establish a causal relationship between the socioeconomic factors studied and MA. On the other hand, the ethnic, racial, and socioeconomic diversity of the sample and the pioneering nature of the data stand out.

MA is an important parameter of pubertal development that can reflect socioeconomic and nutritional changes in a population. The anticipation of this event, which has been described over several decades in Brazil and around the world, has been attributed, in large part, to the increasing prevalence of obesity in childhood and adolescence. Earlier MA is associated with a higher risk of cardiovascular and metabolic diseases in adulthood.

In summary, in the state of Amazonas, where MA and its association with socioeconomic and nutritional factors were previously unknown, this study showed that, regardless of socioeconomic status or ethnicity, excess weight was associated with earlier menarche. Presenting this data can help public policies combat modifiable risk factors, such as being overweight. Furthermore, helping to compose the panorama of MA in this country of continental dimensions and extensive ethnic, cultural, and socioeconomic diversity that is Brazil.

## Conflicts of interest

The authors declare no conflicts of interest.

## Appendix Supplementary materials

Image, application 1

## References

[1] Herman-Gidens M.E. (2006). Recent data on pubertal milestones in United States children: the secular trend toward earlier development. Int J Androl.

[2] Feibelmann T.C., Silva A.P., Resende D.C., Resende E.A., Scatena L.M., Borges M.D. (2015). Puberty in a sample of Brazilian schoolgirls: timing and anthropometric characteristics. Arch Endocrinol Metab.

[3] Kaplowitz P.B. (2008). Link between body fat and the timing of puberty. Pediatrics.

[4] Sørensen K., Mouritsen A., Aksglaede L., Hagen C.P., Mogensen S.S., Juul A. (2012). Recent secular trends in pubertal timing: implications for evaluation and diagnosis of precocious puberty. Horm Res Paediatr.

[5] McDowell M.A., Brody D.J., Hughes J.P. (2007). Has age at menarche changed? Results from the National Health and Nutrition Examination Survey (NHANES) 1999-2004. J Adolesc Health.

[6] Anderson S.E., Dallal G.E., Must A. (2003). Relative weight and race influence average age at menarche: results from two nationally representative surveys of US girls studied 25 years apart. Pediatrics.

[7] Anderson S.E., Must A. (2005). Interpreting the continued decline in the average age at menarche: results from two nationally representative surveys of US girls studied 10 years apart. Pediatrics.

[8] Biro F.M., Pajak A., Wolff M.S., Pinney S.M., Windham G.C., Galvez M.P. (2018). Age of menarche in a longitudinal US cohort. J Pediatr Adolesc Gynecol.

[9] Aksglaede L., Sørensen K., Petersen J.H., Skakkebaek N.E., Juul A. (2009). Recent decline in age at breast development: the Copenhagen Puberty Study. Pediatrics.

[10] Kac G., Coelho M.A., Velasquez-Melendez G. (2000). Short report: secular trends in age at menarche for women born between 1920 and 1979 in Rio de Janeiro. Brazil. Ann Hum Bio..

[11] Castilho S.D., Pine C.D., Bento C.A., Barros-Filho A.A., Cocetti M. (2012). Secular trends in age at menarche in relation to body mass index. Arch Endocrinol Metab.

[12] Silva H.P., Padez C. (2006). Secular trend in age at menarche among Caboclo populations from Pará, Amazonia, Brazil: 1930-1980. Am J Hum Biol.

[13] Gemelli I.F., Farias E.D., Souza O.F. (2016). Age at menarche and its association with excess weight and body fat percentage in girls in the southwestern region of the Brazilian. J Pediatr Adolesc Gynecol.

[14] Instituto Brasileiro de Geografia e Estatística - IBGE. Características Étnico-raciais da População - um estudo das categorias de classificação de cor ou raça; 2011. 95 pp.

[15] Barros B.S., Kuschnir M.C., Bloch K.V., Silva T.L. (2019). ERICA: age at menarche and its association with nutritional status. J Pediatr (Rio J).

[16] Song Y., Ma J., Wang H., Wang Z., Hu P., Zhang B. (2014). Trends of age at menarche and association with body mass index in Chinese school-aged girls, 1985-2010. J Pediatr.

[17] Gemelli I.F., Farias E.D., Spritzer P.M. (2020). Association of body composition and age at menarche in girls and adolescents in the Brazilian Legal Amazon. J Pediatr (Rio J).

[18] Lee J.M., Appugliese D., Kaciroti N., Corwyn R.F., Bradley R.H., Lumeng J.C. (2007). Weight status in young girls and the onset of puberty. Pediatrics.

[19] He Q., Karlberg J. (2001). BMI in childhood and its association with height gain, timing of puberty, and final height. Pediatr Res.

[20] Lyu Y., Mirea L., Yang J., Warren R., Zhang J., Lee S.K. (2014). Secular trends in age at menarche among women born between 1955 and 1985 in Southeastern China. BMC Women's Health.

[21] Martin M.A., Valeggia C. (2018). Timing of pubertal growth and menarche in indigenous Qom girls of Argentina. Ann Hum Biol.

[22] Figueroa Sobrero A., Evangelista P., Kovalskys I., Digón P., López S., Scaiola E. (2016). Cardio-metabolic risk factors in Argentine children. A comparative study. Diabetes Metab Syndr.

[23] Benefice E., Luna Monrroy S.J., Lopez Rodriguez R.W., Ndiaye G (2011). Fat and muscle mass in different groups of pre-pubertal and pubertal rural children. Cross-cultural comparisons between Sahelian (rural Senegal) and Amazonian (Beni River, Bolivia) children. Ann Hum Biol..

[24] Jansen E.C., Herrán O.F., Villamor E. (2015). Trends and correlates of age at menarche in Colombia: results from a nationally representative survey. Econ Hum Biol.

[25] Pereira A., Corvalan C., Merino P.M., Leiva V., Mericq V. (2019). Age at pubertal development in a Hispanic-Latina female population: should the definitions be revisited?. J Pediatr Adolesc Gynecol.

[26] Bubach S., Menezes A.M., Barros F.C., Wehrmeister F.C., Gonçalves H., Assunção M.C. (2016). Impact of the age at menarche on body composition in adulthood: results from two birth cohort studies. BMC Public Health.

[27] Tavares C.H., Haeffner L.S., Barbieri M.A., Bettiol H., Barbieri M.R., Souza L. (2000). Age at menarche among schoolgirls from a rural community in Southeast Brazil. Cad Saude Publica.

[28] Junqueira Do Lago M., Faerstein E., De Souza Lopes C., Werneck G.L., Pró-Saúde Study (Rio de Janeiro, Brazil) (2003). Family socio-economic background modified secular trends in age at menarche: evidence from the Pró-Saúde Study (Rio de Janeiro, Brazil). Ann Hum Biol.

[29] Dreyfus J., Jacobs D.R., Mueller N., Schreiner P.J., Moran A., Carnethon M.R. (2015). Age at menarche and cardiometabolic risk in adulthood: the coronary artery risk development in young adults study. J Pediatr.

[30] Zhang Z., Hu X., Yang C., Chen X. (2019). Early age at menarche is associated with insulin resistance: a systemic review and meta-analysis. Postgrad Med.

