# Supplementary figures and images for: Nutritional status and age at menarche in Amazonian students
Source: J Pediatr (Rio J). 2024 Mar 21;100(4):406–12. doi: 10.1016/j.jped.2024.03.002 (PMC11331225; doi:10.1016/j.jped.2024.03.002)

JPED-D-23-00515 – Supplementary Material

**Supplementary Figure 1- Sample selection process:**


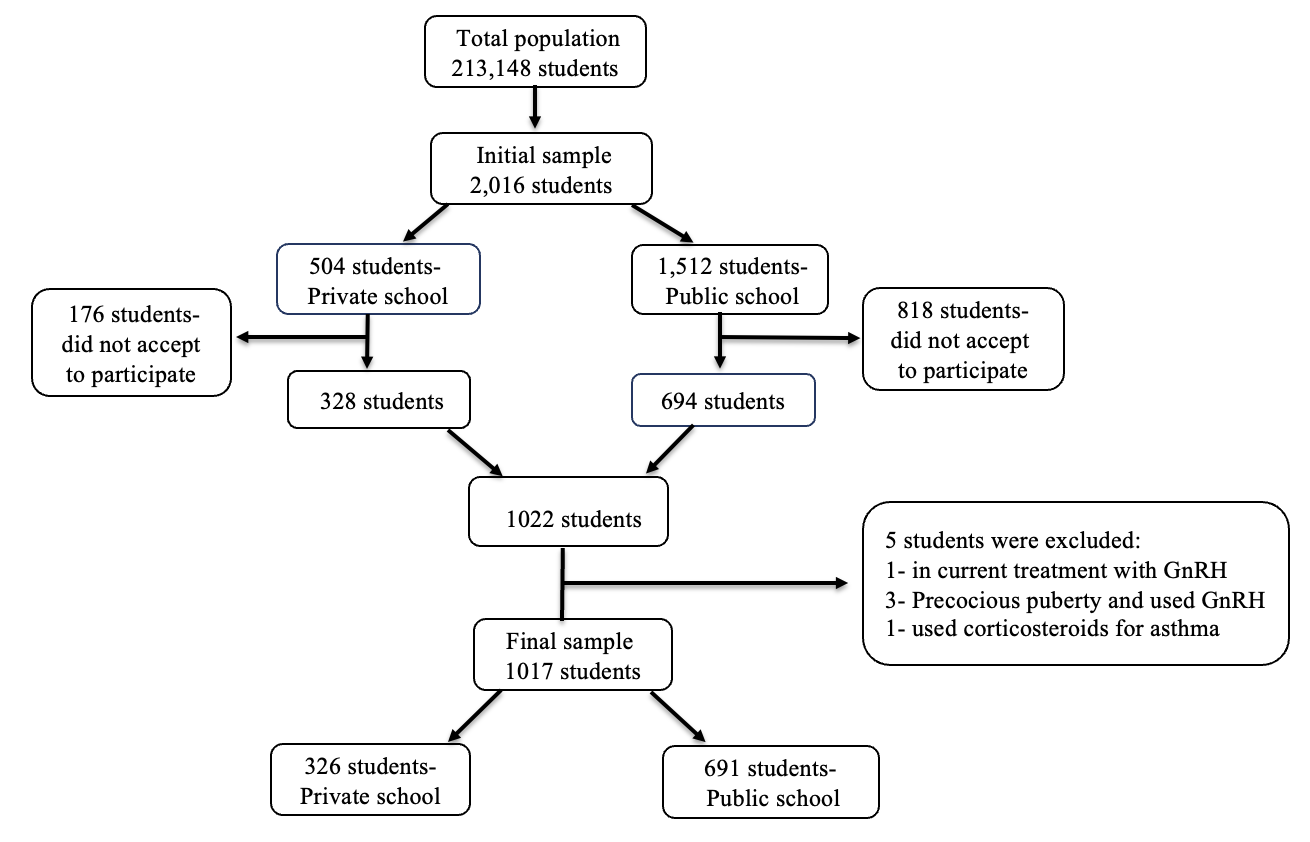

Supplement: Supplementary file 1 [file mmc1.docx]
